# Sedentary behaviour among elite professional footballers: health and performance implications

**DOI:** 10.1136/bmjsem-2015-000023

**Published:** 2015-07-17

**Authors:** Richard Weiler, Daniel Aggio, Mark Hamer, Tom Taylor, Bhavesh Kumar

**Affiliations:** 1 University College London Hospitals NHS Foundation Trust and UCL Institute of Sport, Exercise and Health, London, UK; 2 University College London, UCL-PARG (University College London Population Health Domain Physical Activity Research Group), London, UK; 3 Department of Sports Medicine and Sports Science, West Ham United Football Club, Essex, UK; 4 The FA Centre for Disability Football Research, Burton-Upon-Trent, UK; 5 Department of Epidemiology and Public Health, University College London, London, UK; 6 Applied Science, College of Engineering, Swansea University, Wales, UK

## Abstract

**Background:**

Elite athletes should have little concern about meeting recommended guidelines on physical activity. However, sedentary behaviour is considered a health risk independent of physical activity, and is recognised in public health guidelines advising against prolonged sedentary time. There has been very little research on athletes’ physical activity behaviour outside elite sport.

**Methods:**

Given health and performance links, we investigated in-season post-training activity levels in 28 elite professional footballers during the English Premiership season. Players volunteered to wear a triaxial wrist accelerometer for 1 week, removing it only for training and matches. In total, 25 players met the inclusion criteria for analysis. Players recorded on average 632.6 min wear time p/day during the post-training period (SD±52.9) for a mean of 3.8 days (SD±1.5).

**Results:**

On average, players recorded 76.2 min p/day (SD±28.8) of moderate or vigorous activity post-training. The majority (79%) of post-training time was spent in sedentary activities (500.6 min per day±59.0).

**Conclusions:**

Professional footballers are alarmingly sedentary in their leisure time, and comparatively more so than non-athletic groups of a similar age and older. This raises questions over optimum recovery and performance, as well as long-term health and cardiovascular risk. Worryingly, retirement from elite sport is likely to further imbalance activity and sedentary behaviour. Promoting regular periodic light to moderate leisure time activity could be beneficial. Further research and provision of education and support for players is required in this area.

## Background

Elite athletes competing in physically demanding sports should have little concern about meeting recommended guidelines on physical activity to accrue health benefits. However, sedentary behaviour is now considered a health risk factor independent of physical activity time,^1–5^ and is recognised in updated public health guidelines that advise against prolonged sedentary time.^6^

There has been very little research on athletes’ physical activity behaviour outside elite sport. In the short term, athletes’ lifestyles may compromise their recovery and performance. In the long term, poor lifestyles may pose health risks after the athlete has retired.

Sedentary behaviour has been defined as any waking behaviour characterised by an energy expenditure under 1.5 metabolic equivalents (MET; one MET is the same as 1 kcal/kg/hour and is roughly equivalent to the energy cost of not moving) while in a sitting or reclining posture.^7^ In adults, excessive sitting has been independently associated with adverse health, including abnormal glucose tolerance and type 2 diabetes, the metabolic syndrome, obesity, cardiovascular disease and mortality.^2^ ^8^ ^9^

Epidemiological studies have demonstrated a correlation between sedentary time and health risk.^10–12^ Every 2 h of daily television viewing has been associated with a 20% increased risk of type 2 diabetes, and 13% increased risk of all-cause mortality, independently of physical activity.^13^ ^14^ Sitting for more than 10 h per day has been associated with higher body mass index and waist circumference, systolic and diastolic blood pressure, total serum cholesterol, triglycerides and non-fasting glucose levels, compared to those reporting a total sitting time of less than 4 h per day.^15^ In a controlled trial, replacing sitting with just 2 min of walking every 20 min was shown to reduce serum insulin and postprandial blood glucose by 24%, and lower blood pressure by a mean of 2–3 mm Hg.^16^ These findings suggest that adults should be encouraged to reduce their daily total sedentary time.

**What are the new findings**

- Whilst exceeding recommended levels of physical activity for health and fitness, professional footballers spend the majority of their leisure time in sedentary activity.
- The majority (79%) of waking hours (excluding training/matches) was spent sedentary (500.6 min±59.0 per day).
- These levels of sedentariness are far greater than comparable data published on non-athlete and athlete samples of a similar age and body mass index.

**How might it impact on clinical practice in the near future**

- These findings raise important questions over leisure time management for optimum recovery, performance and perhaps reducing injury risk.
- There is a likelihood of imbalance between physical activity and sedentariness following retirement, which, coupled with the possibility of a less athletic diet and increasing age, poses mounting risk factors for chronic disease.
- Interventions could be provided by club medical staff and via professional sports governing bodies or athlete associations.
- This study indicates a necessity to explore objective sedentary behaviour profiles and optimal recovery more widely and deeply in athletes.

The benefits of light intensity activity (1.6–3.0 METs) such as standing and gentle walking are gaining interest, having been shown to improve blood glucose profiles,^17^ as well as lipoprotein lipase activity and therefore triglyceride and high-density lipoprotein cholesterol levels, independently of moderate and vigorous physical activity.^18^ Genetics studies on muscle biopsy tissue have shown that compared with uninterrupted sitting, activity bouts favourably promote the expression of proteins modulating anti-inflammatory and anti-oxidative pathways.^19^ The procoagulant effects of prolonged sitting can also be ameliorated.^20^

Excessive sedentary time is also likely to have implications on recovery and therefore sport performance. Muscle soreness following increased or unaccustomed exercise has been linked with reduced strength and performance.^21^ Studies involving short bouts of high intensity exercise, making them applicable to many sports including football, have shown that active recovery strategies, including light and moderate intensity activity, appear favourable when compared with passive (sedentary) recovery. 22 23 Relative rest is necessary for recovery, but the impact of prolonged sedentariness on recovery and therefore athletic performance or injury risk is poorly understood.

Given these causes for concern, and the dearth of objective data on the physical activity behaviour of elite athletes outside of sport, we investigated post-training (or leisure-time) physical activity behaviour in elite professional footballers during the English Premiership football season.

## Methods

### Participants

As part of ongoing monitoring of the health, well-being and recovery of all first team squad members at an English Premier League football club, individual written informed consent was obtained from 28 training, non-injured players to objectively monitor their physical activity levels during the 2014–2015 English Premier League season.

### Physical activity assessment

Each player was instructed to wear a validated *GENEActiv* triaxial wrist accelerometer^24^ for 1 week during the 2014/2015 season, removing it only for club training and matches. The device provides a measure of the intensity, frequency and duration of physical activity. Data were collected at a frequency of 50 Hz.

### Data analysis

We used previously defined cut-off points to calculate daily times in each activity intensity band.^24^ Activity intensity was categorised as sedentary activity (<1.5 MET), light (1.5–3 MET), moderate (3–6 MET) and vigorous (>6 MET). Non-wear time was defined as intervals of at least 120 consecutive minutes of zero movement. Time (hours) spent in each activity intensity was calculated for every valid day of data recorded, defined as at least 500 min of continuous wear time during waking hours of the post-training period. Data classified as time in bed were excluded from analyses. Summary statistics were generated using SPSS V.22.

## Results

A total of 25 participants provided valid accelerometry data, with mean age 26.8 years (SD±4.4), height 184 cm (SD±7.1), weight 81.9 kg (SD±7.8) and body mass index (BMI) 23.9 (SD±1.3). Participants wore the device for a mean of 3.8 days (SD±1.5), with 80% wearing the device for three or more days. Mean wear time was 632.6 min wear time per day (SD±52.9).

A summary of the physical activity data is provided in table 1.

**Table 1:** Summary of post-training daily activity levels in premiership football players (n=25)

Variable	Mean±SD	Mean % waking hours of leisure time
Valid days worn	3.8±1.5	–
Average GeneActiv wear time (min/day)	632.6±52.9	–
Sedentary time in waking hours (min/day)	500.6±59.0	79
Light activity (min/day)	55.7±28.8	9
Moderate activity (min/day)	74.1±28.1	12
Vigorous activity (min/day)	2.1±3.9	<1

## Discussion

To the best of our knowledge, this is the first published study providing objective insight into the physical activity and inactivity behaviours of elite footballers away from training and competition.

In our sample of elite professional footballers, we observed that a high percentage of their waking non-training hours was spent in a sedentary state (79%) in activities requiring less than 1.5 METS, such as sitting, lying down, watching television, passive transportation, using a computer or playing video games.^25^ It is therefore clearly possible to meet and exceed physical activity recommendations, but spend large parts of the day sedentary.

While not accounting for occupational training and competition activity and therefore not directly comparable, this level of sedentariness is still far greater than data published on non-athlete samples of a similar age and BMI, which generally report 50–60% of waking hours spent in sedentary time.^26^ Elderly populations have also been found to spend less time being sedentary.^27^ A recent study found that non-football athletes spend 7.7 h (SD±2.7) per day sedentary based on subjective self-recall, and this correlated positively with measures of adiposity.^28^

These data suggest that highly motivated professional athletes are not exempt from high levels of sedentary behaviour and modern-day vices that are conducive to a sedentary lifestyle. Indeed, these findings suggest far greater levels of sedentariness than other previously studied populations. Given the genetic, proteomic, metabolomic, physiological and epidemiological evidence available, our findings have led us to question whether such a high proportion of sedentary time is the best method for optimum recovery, optimum performance and longer term health outcomes.

Perhaps of most concern is the likelihood of further imbalance between physical activity and sedentariness following retirement, should these behaviours continue. Coupled with the possibility of a less athletic diet and increasing age, risk factors for chronic disease may begin to mount. Fitness, which is an important health determinant in its own right,^29^ declines rapidly with deconditioning and age, and there is evidence that added life expectancy is only likely if physical activity is maintained after retirement from elite competition.^30^ Thus, having previously been a fit athlete does not necessarily confer long-term protection to cardiometabolic health.

To date, there are no specific recommendations on breaking up sedentary time, particularly for elite athletes, and more evidence is required to establish firm guidelines. The current available evidence suggests that during waking hours, breaking up sitting every 20 min by standing up or walking may be beneficial for cardiometabolic health, although further evidence is required to establish firm recommendations.

Delivering accurate and effective lifestyle advice to professional athletes to help establish favourable patterns of physical activity behaviours could benefit their recovery and performance while in their profession and importantly improve health in the long term after retirement from professional sport. Primary targets could include athletes coming up to retirement, those who are not involved in regular competitive action, as well as athletes who show evidence of greater than average detriments to fitness and body composition following the off season.

Interventions could be provided by club medical staff and via professional sports governing bodies or players’ associations. They should be tailored and based on the athlete's contextual information, such as family, local environment or modes of transport available. Movement sensors such as the device used in the present study can be useful in monitoring and providing feedback to players. Our data have allowed the participants to implement and test changes to their lifestyles, using adapted rest and recovery strategies, lifestyle behaviour change strategies and ongoing feedback systems.

This study indicates a necessity to explore sedentary behaviour profiles more widely and deeply in athletes. Our pilot study was limited to elite male professional footballers in one club in the English Premier League; thus, we are unable to generalise our results to other athletes, sports, females and different populations. There may be variability in sedentary behaviour between sports, and other sociobiological and environmental determinants, which could include demographics such as cohabitants, ethnicity, culture and socioeconomic strata. Future work will analyse correlations between leisure-time activity patterns on injury risk and performance parameters, and also look at a more detailed analysis of periods of unbroken sedentary time.

## Conclusions

Professional footballers spend the majority of their leisure time in sedentary activity, raising questions over optimum recovery, performance and perhaps injury risk, as well as long-term health. Worryingly, retirement from elite sport is likely to further imbalance activity and sedentary behaviour. Promoting regular periodic light to moderate leisure time activity could be beneficial. We advocate further research and wider provision of education and support for footballers in lifestyle management.
